# Quality of Life instruments and their psychometric properties for use in parents during pregnancy and the postpartum period: a systematic scoping review

**DOI:** 10.1186/s12955-022-02011-y

**Published:** 2022-07-09

**Authors:** Malene Brekke, Rigmor C. Berg, Amin Amro, Kari Glavin, Trude Haugland

**Affiliations:** 1 VID Specialized University, Oslo, Norway; 2 Norwegian Institute of Public Health, Oslo, Norway; 3 The University of Tromsø, Tromsö, Norway

**Keywords:** Quality of Life, Psychometric properties, Pregnancy, Postpartum, Scoping review

## Abstract

**Purpose:**

To identify instruments used to measure parents’ Quality of Life (QoL) during pregnancy and the postpartum period, and to describe their characteristics and psychometric properties.

**Methods:**

For this scoping review we conducted systematic literature searches in MEDLINE, EMBASE, PsychINFO, CINAHL and HaPI in mid-December 2020, to identify studies evaluating psychometric properties. The COnsensus-based Standards for the selection of health Measurement INstruments (COSMIN) were used to define and categorize psychometric properties. Two reviewers screened the studies independently, and customized screening questions were used to assess eligibility against inclusion criteria. Data were systematically extracted into a predesigned data charting matrix, and descriptively analyzed.

**Results:**

The searches identified 5671 studies, of which 53 studies met the inclusion criteria. In total, there were 19 QoL instruments: 12 generic and seven period specific. The most reported instruments were SF-36, SF-12 and WHOQOL-BREF, and the most evaluated instruments were SF-12, WHOQOL-BREF, QOL-GRAV, and PQOL. We found that none of the identified instruments had been evaluated for all nine psychometric properties recommended by the COSMIN. The most reported psychometric properties were internal consistency and structural validity. The instruments were primarily assessed in parents residing in Asia (50%), and 83% of the studies were conducted from 2010 to 2020. Only three studies included psychometric measures assessed on fathers.

**Conclusion:**

Our review shows there is extensive evidence on the internal consistency and structural validity of QoL instruments used on parents during pregnancy and the postpartum period, but that the evidence on other psychometric properties is sparse. Validation studies and primary studies are needed to provide evidence on the reliability, validity, responsiveness, and interpretability of QoL instruments for this target group, in particular for fathers and partners.

**Supplementary Information:**

The online version contains supplementary material available at 10.1186/s12955-022-02011-y.

## Introduction

Quality of Life (QoL) is a widely used outcome in health care research. However, there is not one agreed definition or measurement of QoL and researchers have argued that QoL is an ambiguous concept [1–3]. The World Health Organization (WHO) defines QoL as: “… individuals’ perceptions of their position in life in the context of the culture and value systems in which they live and in relation to their goals, expectations, standards, and concerns” [4]. This definition embraces the subjective aspects of the concept, which is an agreed perception in the measurement of QoL [5–7]. In addition, QoL is considered to be a multidimensional construct [5], and identification of dimensions of importance for QoL has to a great extent been achieved [2]. The most common dimensions used in health care research are physical, psychological, and social functions [5].

A number of QoL instruments have been designed to fit specific situations of life and health [5, 8], such as parenting. The transition to parenthood involves major changes both psychologically and physically [9, 10], and several instruments have been used or developed to measure generic and specific QoL during pregnancy and the postpartum period [11, 12]. The construct, concept, and content of these QoL instruments and their dimensions must be tested by statistical and psychometric analyses to determine and confirm the instrument’s suitability to the target group [5, 13, 14]. The COnsensus-based Standards for the selection of health Measurement INstruments (COSMIN) has provided a taxonomy for evaluation of psychometric properties of measurement instruments [15]. COSMIN distinguishes between three domains of psychometric properties, which includes nine recommended properties. The domain reliability contains the properties internal consistency, reliability, and measurement error. The domain validity contains content validity, criterion validity and the three properties of construct validity – structural validity, cross-cultural validity and hypotheses-testing. The domain responsiveness only includes the psychometric property responsiveness. COSMIN also includes interpretability which refers to what the scores on an instrument mean. Interpretability is not a psychometric property as it does not refer to the quality of an instrument [15, 16]. The psychometric properties of an instrument should be confirmed to be adequate. Otherwise, there is a chance of imprecise and biased results that may lead to wrong conclusions [17]. Although an instrument can never be proven valid, credible evidence from multiple studies can show that it is sensible and useful for its intended purpose [5].

Existing literature reviews regarding QoL in pregnant and postpartum populations tend to focus on factors associated with QoL [18–21] and identification of generic or disease/period specific QoL instruments [11, 12, 18, 22], rather than the psychometric evaluations of the instruments. However, a literature review with the secondary objective to evaluate existing pregnancy and postpartum period specific QoL instruments, by Mogos et al. in 2013 [11], reported psychometric properties for three period specific QoL instruments. The instruments were the Maternal Postpartum Quality of Life Questionnaire (MAPP-QOL), Mother Generated Index (MGI), and Rural Postpartum Quality of Life (RPQOL). Mogos et al. reported that there were few instruments designed specifically for the general maternity care setting, and that instruments specific for the maternal population are too narrow and do not include crucial aspects of reproductive health related to women’s QoL. However, the literature review of Mogos et al. neither reports a systematic search strategy, includes psychometric evaluations of generic instruments for this specific population, nor distinguishes between QoL instruments specific for the pregnant/postpartum period and instruments specific for maternal populations or female conditions. Furthermore, most literature reviews on QoL in the pregnant and postpartum period focus on the maternal population only [11, 12, 19–22]. Health care research on men’s transition to fatherhood [23–29] shows that this period affects their mental health and emotional wellbeing, the need for support and the fatherhood identity. These results support the need to include the paternal aspects of QoL during this period of life, and in turn, instruments that are validated and reliable in both fathers as well as mothers.

Scoping reviews are exploratory and descriptive in nature, and useful to determine the value of undertaking a full systematic review [30]. To the best of our knowledge, there are no systematic reviews that evaluate QoL instruments and their psychometric properties in the general population of mothers and/or fathers during pregnancy and the postpartum period. Scoping the literature with the aim of identifying and describing such QoL instruments can provide useful descriptive information on QoL instruments used in this target group and identify research gaps that can provide recommendations for future research, for both primary studies and systematic reviews [30]. In addition, the results of a scoping review may be useful for the selection processes of QoL instruments in future health care research [31]. Therefore, this systematic scoping review aims to identify instruments used to measure mothers’ and/or fathers’ QoL during pregnancy and the postpartum period, and to describe their characteristics and psychometric properties.

## Methods

### Protocol and reporting

We developed a protocol in line with the methodological framework by Arksey and O’Malley [30], later revised by Levac et al. [32]. The protocol is available through ResearchGate [33]. There were no deviations from the protocol, and we report the review results in accordance with the Preferred Reporting Items for Systematic reviews and Meta-Analyses extension for Scoping Reviews (PRISMA-ScR) [34].

### Eligibility criteria

Given the aim of the review, the main inclusion criterion was that the study described one or more instruments measuring QoL in mothers and/or fathers, also referred to as parents, during pregnancy and/or the postpartum period and gave information on one or more psychometric properties of the instrument. The eligibility criteria are specified in Table 1.

**Table 1:** Eligibility criteria

	Inclusion and exclusion criteria
Population	Mothers and/or fathers during pregnancy and the postpartum period up to 12 months post birth. Studies with parents with specific conditions related to pregnancy or the postpartum period were included when we considered the condition common to the pregnant and/or postpartum population, e.g. mild/moderate nausea and vomiting, pelvic floor and/or back pain, tear during birth. There were no restrictions regarding the parents' age, ethnicity, or residence, or the health care setting We excluded studies in which more than 25% of the sample were parental subpopulations. Parental sub-population was defined as parents with, or parents of children with, a health-related diagnosis (e.g. cancer, HIV, heart failure, organ transplant, diabetes, incontinence) or specific life situation (e.g. violence, abuse, bullying). If psychometric properties were reported separately for the sub-population and the healthy population, the study was included
Instrument	We operationalized QoL instruments as generic or specific instruments developed to collect data on QoL, and we understood QoL as a subjective and multidimensional construct, as described in the introduction [4, 5] We excluded studies of instruments specifically developed to identify QoL in a parental sub-population. Measurements of interrelated concepts such as satisfaction with life and well-being were excluded, as were studies that lacked or incorrectly referenced the original developer of the QoL instrument being reported. The latter exclusion criterion was because of such studies’ inability to report on information important for our understanding of which instrument was used in their study
Outcome	Psychometric properties. We understood psychometric properties as measurements of reliability, validity, responsiveness and/or interpretability as defined by COSMIN [15]. When multiple publications reported identical measures of psychometric properties from the same study and were based on the same population, we included the study that had the most comprehensive reporting of psychometric properties
Study design	We included studies of any design as long as it reported a psychometric evaluation of QoL
Language	English and Scandinavian languages
Year	Publications dating 1990–2020

### Information sources and search strategy

A systematic search of the literature was conducted in mid-December 2020, in the databases MEDLINE, EMBASE, PsychINFO, CINAHL, and HaPI (Health and Psychosocial Instruments). The search was limited to records from 1990 to December 2020. The complete search is shown in Additional file 1.

The search strategy was developed and performed by a librarian experienced in systematic searches of scientific databases, in cooperation with the reviewers. In addition, one reviewer (MB) screened the reference lists of all included studies and relevant systematic reviews for relevant studies not identified in our database search.

### Study selection

We imported the records identified in the database searches to EndNote [35] and then to the screening tool Rayyan [36]. We searched for and deleted duplicate references in both programs. We performed study selection in two stages. First, using Rayyan, we screened all titles and abstracts, and in the second step, the full text of studies deemed eligible in the first step. At both stages, screening was done by pairs of reviewers (MB and TH/RB/AA/KG), independently, and disagreements were solved through consensus. A third reviewer (RB/TH) was involved in the final selection of three studies when the review pair was unsure of inclusion. We used customized screening questions at both stages to assess eligibility against the inclusion criteria and only studies that both reviewers agreed met all inclusion criteria were included. The screening questions were yes/no questions based on the eligibility criteria for the population, period of measurement, sub-populations, type of instrument and psychometric properties (see Table 1). For example: Is the population women and/or men during pregnancy or postpartum period?

### Data charting process and data items

One reviewer (MB) systematically extracted data (variables) from the included sources into a pre-designed data charting matrix in Excel to enable consistency. The extracted data was controlled for accuracy and completeness by a second reviewer (TH/RB/AA/KG). A third reviewer (RB/TH) was consulted to reach a final consensus on the extraction of psychometric properties of two studies. The data charting matrix included characteristics of published studies (publication year, country, design), study objectives, recruitment, data collection, sample, instrument characteristics (name, developer, items, dimensions, scoring), and psychometric evaluation (reliability, validity, responsiveness, and interpretability) as defined by COSMIN [15].

We did not assess studies’ risk of bias because that is not a prerequisite in scoping reviews [30, 32]. Consequently, we did not categorize the psychometric properties from each study as sufficient/insufficient/indeterminate, as this requires methodological quality assessments of the included studies. Additionally, the aim of this scoping review was to describe, not evaluate, the psychometric properties of the included instruments. We note selected limitations of the included studies, relevant for further research, in the results and discussion sections.

### Synthesis of results

By charting each study and compiling the data in a single spreadsheet, we could group the variables according to their chief characteristics and carry out descriptive analyses by using frequencies and cross-tabulations. The grouping included sorting the variables and summarizing the extracted data in tables (Tables 2, 3, 4) and text.

## Results

**Fig. 1:**
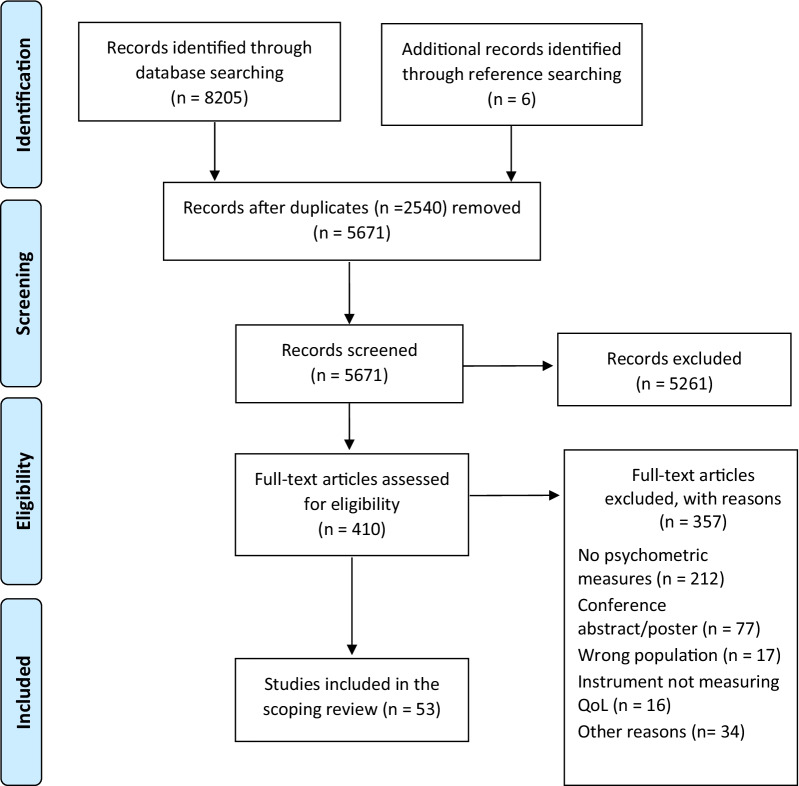
Flow diagram of literature search

The flow diagram in Fig. 1 shows that the search returned 5671 unique records, of which 410 were eligible for full-text screening. We included 53 studies, one of which was identified from the search in the reference lists.

### Study characteristics

Characteristics of the included studies are described in Table 2. The studies were published between 2002 and December 2020, with 51% published after 2014. Most publications (n = 52/98%) were journal articles, but we also included one dissertation. All were in English. The studies were conducted in 21 different countries, with 49% (n = 26) of the studies being from Asia, 26% (n = 14) from Europe, 11% (n = 6) from North America, and the remaining 13% (n = 7) from South America, Africa, and Oceania. Table 2 shows the countries where the studies were conducted. The ‘other’ countries is one study each from Bangladesh, Canada, Czech Republic, Germany/Switzerland, Hungary, Jordan, Malaysia, and Spain. The sample size in the included studies ranged from 30 to 5079 participants, with a mean of 507 participants. There were no studies conducted on only fathers, and only four studies (7%) included couples [25, 37–39]. About half of the studies assessed QoL during pregnancy and half during the postpartum period.

**Table 2:** Characteristics of the included studies

	Number of studies N = 53
	n (%)
Publication year	
1990–1999	0 (0)
2000–2004	1 (1.9)
2005–2009	8 (15.1)
2010–2014	17 (32.1)
2015–2019	21 (39.6)
2020	6 (11.3)
Country	
Iran	8 (15.1)
China	6 (11.3)
USA	5 (9.4)
Australia	3 (3.0)
Japan	3 (5.7)
Portugal	3 (5.7)
Scotland	3 (5.7)
Turkey	3 (5.7)
Taiwan	3 (5.7)
Brazil	2 (3.8)
Malawi	2 (3.8)
Netherlands	2 (3.8)
UK	2 (3.8)
Other countries	8 (15.1)
Design	
Cross-sectional	21 (39.6)
Longitudinal	15 (28.3)
Validation	17 (32.1)
Participants	
1–49	3 (5.7)
50–99	5 (9.4)
100–299	21 (39.6)
300–499	9 (17)
500–999	9 (17)
1000+	6 (11.3)
Gender	
Women	49 (92.5)
Men/partners	0 (0)
Women + men/partners (couples)	4 (7.5)
Measurement timepoint/period	
Pregnancy	20 (37.7)
Postpartum (< 12 months)	24 (45.3)
Pregnancy + postpartum	9 (17)
QoL Instruments*	
*Generic instruments*	**40 (70.2)**
Short Form 36-item health survey (SF-36)	10 (17.5)
RAND 36-item health survey (RAND-36)	1 (1.8)
Short Form 12-item health survey (SF-12)	9 (15.8)
Short Form 8-item health survey (SF-8)	1 (1.8)
World Health Organization Quality of Life Questionnaire Brief version (WHOQOL-BREF)	9 (15.8)
EUROHIS-QoL-8	1 (1.8)
Quality of life scale (QOLS)	1 (1.8)
Nottingham Health Profile (NHP)	1 (1.8)
EQ-5D-3L + EQ VAS	2 (3.6)
Patient reported outcomes measurement information system-43 (PROMIS 43)	1 (1.8)
Patient reported outcomes measurement information System-global short form (PROMIS GSF)	3 (5.3)
Duke health profile (DUKE)	1 (1.8)
*Specific instruments*	**17 (29.8)**
Maternal quality of life Index (M-QLI)	1 (1.8)
Mother generated Index (MGI)	6 (10.5)
Quality of life Gravidarum (QOL-GRAV)	3 (5.3)
Maternal postpartum quality of life questionnaire (MAPP-QOL)	2 (3.5)
Rural postpartum quality of life (RPQOL)	1 (1.8)
Postpartum quality of life (PQOL)	3 (5.3)
Short form postpartum quality of life (SF-PQOL)	1 (1.8)

Bold indicates the summary of generic/specific instruments

*57 observations in 53 studies

Of the 53 studies, 17 (32%) were validation studies. Six (35%) of these were from Europe [40–45], five (29%) from Asia [46–50], three (18%) from North America [51–53], two (12%) from Oceania [54, 55], and one (6%) from South America [56]. Five (29%) of the studies concerned Mother Generated Index (MGI) [42–44, 46, 56]. There were two (12%) studies on Quality of Life Gravidarum (QOL-GRAV) [45, 47], two on Postpartum Quality of Life (PQOL) [49, 50] and two on Patient Reported Outcomes Measurement Information System Global Short Form (PROMIS-GSF) [53, 54]. Short Form Postpartum Quality of Life (SF-PQOL) [48], World Health Organization Quality of Life Questionnaire Brief version (WHOQOL-BREF) [55], Nottingham Health Profile (NHP) [51], Maternal Postpartum Quality of Life Questionnaire (MAPP-QOL) [52], Short Form 36-item Health Survey (SF-36) [41], and EQ-5D-3L [40] were addressed in one study each (6%).

### Identified Quality of Life instruments

The included studies described a total of 19 different QoL instruments (Tables 2, 3, 4), of which 12 (63%) were generic instruments and seven (37%) were specific for QoL measures in pregnancy and/or the postpartum period. Some of the studies reported on multiple instruments, resulting in 57 observations of instruments in the 53 included studies. As shown in Table 2, the 12 generic QoL instruments were evaluated in 40 (70.2%) studies and the most commonly used instruments were SF-36 [40, 41, 57–64], Short Form 12-item Health Survey (SF-12) [65–73], and WHOQOL-BREF [25, 37, 38, 45, 55, 74–77]. Instruments specifically developed for pregnant and/or postpartum populations were evaluated in 17 (29.8%) studies. Of these, the most commonly used instruments were the MGI [42–44, 46, 56, 78], QOL-GRAV [45, 47, 79], and PQOL [48–50]. The majority of the instruments were self-administered questionnaires, but MGI [42–44, 46], SF-36 [64], EQ-5D-3L [80], and Quality of Life Scale (QOLS) [81] were also interviewer-administered.

**Table 3:** Characteristics of the identified instruments*

Instrument full name (Short name)	Period for measurement	Evaluated in following studies:	Language evaluated in	Number of items (domains)	Scored by	Domains/subscales	Interpretation of scores
[reference to instrument developer**] Version		Author, publication year					
*Generic instruments (n = 12)*							
**Short Form 36-item Health Survey (SF-36)** [82–85] All versions	Pregnancy	Alzboon and Vural, 2019[57], Jomeen and Martin, 2005[41], Kugahara and Ohashi, 2006 [58], Li et al., 2012[61], RezaeiNiaraki et al., 2019[63]	Arabic English (UK) Japanese Chinese Iranian	36 (8/2)	Domain scores Component summary scores	Physical functioning, role-physical, bodily pain, general health, vitality, social functioning, role-emotional, and mental health Two component summaries: physical component summary (PCS-36) and mental component summary (MCS-36)	Higher score indicates better health
	Postpartum	Jansen et al., 2007[40], Lau Wong and Chan, 2008 [59],Leroy and Lopes, 2012 [60], Parsa et al., 2019 [62], Trivino-Juarez et al., 2017 [64]	Dutch Chinese Portuguese Iranian Spanish				
**RAND 36-item Health Survey (RAND-36)** [86] Version 1.0	Pregnancy	Dalmida et al., 2010 [87]	Spanish	36 (8/2)	Domain scores Component summary scores	Physical functioning, role-physical, bodily pain, general health, vitality, social functioning, role-emotional, and mental health Two component summaries: physical component summary (PCS) and mental component summary (MCS)	Higher score indicates better health
**Short Form 12-item Health Survey (SF-12)** [88, 89] All versions. Abbreviated version of SF-36	Pregnancy	Bai et al., 2016 [66], Hirose et al., 2020 [69], Lau, 2013 [70], Ngai and Ngu, 2013 [71], Tsai et al., 2016 [73]	Dutch Japanese Chinese Chinese Chinese	12 (8/2)	Component summary scores	Physical functioning, role-physical, bodily pain, general health, vitality, social functioning, role-emotional, and mental health Two component summaries: physical component summary (PCS-12) and mental component summary (MCS-12)	Higher score indicates better health
	Postpartum	Ayers et al., 2018 [65], Desouky Mora and Howell, 2013 [67], Noor and Aziz, 2014 [72]	English (UK) English (USA) Malay				
	Pregnancy + postpartum	Emmanuel and Sun, 2014 [68]	English (Australia)				
**Short Form 8-item Health Survey (SF-8)** [90] Abbreviated version of SF-36	Pregnancy	Nakamura et al., 2018 [91]	Japanese	8 (8/2)	Component summary scores	Physical functioning, role-physical, bodily pain, general health, vitality, social functioning, role-emotional, and mental health Two component summaries: physical component summary (PCS-8) and mental component summary (MCS-8)	Higher score indicates better health
**World Health Organization Quality of Life Questionnaire Brief version (WHOQOL-BREF)** [4, 92] Short version of WHOQOL-100	Pregnancy	Brandão et al., 2020 [37], Daglar, Bilgic, & Ozkan, 2020 [74], Khwepeya et al., 2020 [75], Vachkova et al., 2013 [45]	Portuguese Turkish Chichewa Czech	26 (4)	Domain scores	Physical health (7), Psychological (6), Social relationships (3), Environment (8). Two items on overall QoL and general health	Higher score indicates higher QoL
	Postpartum	Fonseca Nazare and Canavarro, 2012 [38], Khwepeya Monsen and Kuo, 2020 [76], Webster et al., 2010 [55]	Portuguese Chichewa English (Australia)				
	Pregnancy + postpartum	Chen et al., 2019 [25], Mortazavi et al., 2014 [77]	Taiwan Farsi (Iranian)				
**EUROHIS-QoL-8** [93, 94] Abbreviated version of WHOQOL-BREF	Pregnancy + postpartum	Guedes and Canavarro, 2015 [39]	Portuguese	8 (1)	Total score	Overall score of QoL All items represent the four domains of WHOQOL-BREF (physical health, psychological, social relationships, environment)	Higher score indicates higher QoL
**Quality of life Scale (QOLS)** [95, 96]	Postpartum	Akyn et al., 2009 [81]	Turkish	16 (3)	Total score	Relationships and material well-being (5), health and functioning (5), personal, social and community commitment (6)	Higher score indicates higher QoL
**Nottingham Health Profile (NHP)** [97, 98]	Postpartum	Baghirzada Downey and Macarthur, 2013 [51]	English (Canada)	38 + 7 (6)	Domain scores	Part one: Physical mobility (8), social isolation (five), emotional reactions (9), pain (8), sleep (5), energy (3) Part two: seven statements about areas of life	Low score indicates higher QoL
**EQ-5D-3L + EQ VAS** [99, 100]	Postpartum	Jansen et al., 2007 [40], Mahumud et al., 2019 [80]	Dutch Bengali	5 + 1 (5)	Index score and value sets	Mobility, self-care, usual activities, pain/discomfort, anxiety/depression Three severity levels (none, some, extreme/unable to) Sixth item is global evaluation of own health on a visual analogue scale (EQ-VAS)	1 (best state) to -0.594 (worst state) EQ VAS: 0 (worst state) to 100 (best state)
**Patient Reported Outcomes Measurement Information System – 43 (PROMIS-43)** [101–103] Profile version 2.0	Pregnancy	MoghaddamHosseini et al., 2020 [104]	Hungarian	43 (7)	Domain scores	Physical function, Anxiety, Depression, Fatigue, Sleep disturbance, Ability to participate in social roles and activities, Pain interference. (Six items per domain) One item on pain intensity	Higher score represents more of the concept being measured Pain intensity: 0–10
**Patient Reported Outcomes Measurement Information System Global Short Form (PROMIS-GSF)** [105, 106]	Pregnancy	Lundsberg et al., 2018 [53]	English (USA)	10 (2)	Factor scores	Global Physical health (GPH) (4), Global Mental Health (GMH) (4), and two single items on general health and social role	Higher score represents more of the concept being measured
	Pregnancy + postpartum	Slavin et al., 2019 [54]	English (Australia)				
	**Pregnancy + postpartum** (9-item version)	Slavin et al., 2019 [54]	English (Australia)	9 (2)	Factor scores	Physical Health—Pregnancy Postpartum (PH-PP) (5) and Mental Health—Pregnancy Postpartum (MH-PP) (4)	Higher score indicates better physical/ mental health
**Duke Health Profile (DUKE)** [107, 108]	Pregnancy	Wang Liou and Cheng, 2013 [109],	Chinese	17 (5)	Domain scores	Physical, mental, social, general and perceived health	Higher score indicates better health
*Specific instruments (n = 7)*							
**Maternal Quality of Life Index (M-QLI)** Maternal version, partly self-developed as a revised version of the Quality of Life Index	Pregnancy + postpartum	Adams, 2016 [110]	English (USA)	38 + 38 (4)		Health and functioning, psychological and spiritual, social and economic, and family Addition in this maternal version: 4 items on challenges related to motherhood Two parts with identical items. Part one: satisfaction with each item. Part two: level of importance with each item	NA
**Mother Generated Index (MGI)** [43] [42] Antenatal and postnatal version	Pregnancy	Symon and Dobb, 2008 [43], Symon and Dobb, 2011 [44]	English (Scotland)	NA	Index scores	Three-step questionnaire Step 1: specifying up to eight areas of life affected by the pregnancy/having had a baby, and identifies these as positive, negative or neither. Step 2: to give a score from 0 to 10 for each area in step one, based on how the areas have affected the mother over the previous month. Step 3: allocating 20 “spending points” to the areas most important to the responder	NA
	Postpartum	Gomes Ribeiro et al. 2015 [56], , Khabiri et al. 2013, Symon MacDonald and Ruta 2002 [42], Symon and Dobb 2011 [44]	Brazilian-Portuguese Iranian English (Scotland) English (Scotland)				
**Quality of Life Gravidarum (QOL-GRAV)** [45]	Pregnancy	Effati-Daryani et al. 2017 [79], Mirghafourvand et al. 2016 [47], Vachkova et al. 2013 [45]	Persian Persian Czech	9 (1)	Overall score	One domain on pregnancy QoL A two-factor structure have been tested by Mirghafourvand et al., 2016	Lower score indicates higher QoL
**Maternal Postpartum Quality of Life Questionnaire (MAPP-QOL)** [52]	Postpartum	Hill et al. 2006 [52], Gökşin and Ayaz-Alkaya 2018 [111]	English (USA) Turkish	40 (5)	Total score Domain scores	Psychological/baby (9), socioeconomic (9), relational/spouse-partner (5), relational/family-friends (9), health and functioning (8) Two parts. Part one: satisfaction with each item. Part two: level of importance with each item	Highest scores for combinations of high satisfaction/ high importance responses
**Rural Postpartum Quality of Life (RPQOL)** (self-developed, not validated)	Postpartum	Huang et al. 2012 [112]	Chinese	20 (6)	Total score Domain scores	Physical complaints and pain (1), sleep and energy (2), sex satisfaction (3), interpersonal communication (4), self-evaluated living stress (5) and perceived life satisfaction (6)	Lower score indicates higher QoL
**Postpartum Quality of Life (PQOL)** [50]	Postpartum	Zhou et al. 2009 [50], Nikan et al. 2016 [49], Nikan et al. 2018 [48]	Chinese Iranian Iranian	40 (4)	Total score Domain scores	Child care (8), Physical function (12), Psychological function (8), Social support (12)	0 indicates poorest QoL and 100 indicates best QoL
**Short Form Postpartum Quality of Life Questionnaire (SF-PQOL)** Short version of PQOL	Postpartum	Nikan et al., 2018 [48]	Iranian	13 (4)	Total score Domain score	Child care (4), Physical functioning (4), Psychological functioning (3), Social support (2)	0 indicates poorest QoL and 100 indicates best QoL

*In order to describe each of the instruments included we obtained information from the original developer of the instrument

**Reference to original developer of the instrument as found by the authors of this scoping review

Table 3 presents an overview of the characteristics of the 19 QoL instruments, organized by generic and specific instruments, and divided by the three measurement timepoints pregnancy, postpartum, and pregnancy and postpartum. The majority of the instruments measure multiple dimensions, except the MGI, QOL-GRAV, and EUROHIS-QOL-8. The instruments are primarily scored by multiple domain scores, but MAPP-QOL, RPQOL, PQOL and SF-PQOL are scored by both a total score and domain scores. EUROHIS-QoL-8, QOLS and QOL-GRAV are scored by a total score, and EQ-5D-3L by index score. Most instruments operate with the interpretation that high/low scores indicate high/low QoL. However, none of the identified instruments describes a cut-off for low or high QoL.

Nearly all instruments describe dimensions of psychological health, physical health, and social functioning/relationships; QOLS does not mention psychological health, and EQ-5D-3L does not mention dimensions related to social functioning/relationship (Table 3). Moreover, the specific instruments add domains or items of QoL related to pregnancy or the postpartum period. The M-QLI has one domain for challenges related to motherhood, QOL-GRAV has one pregnancy-specific domain which is suggested by the developer as an additional domain to WHOQOL-BREF, and MAPP-QOL, RPQOL, PQOL and SF-PQOL have multiple domains specifically developed to identify QoL in the postpartum period.

### Reported psychometric properties of the instruments

In Table 4 we show which psychometric properties that the studies reported on, according to COSMINs categorization [15]. By far, Cronbach’s alpha was the most commonly reported psychometric property, provided in 43 studies (81.1%), and for all instruments except EQ-5D-3L, Duke Health Profile (DUKE), and MGI. Twenty-five (58%) of these studies reported Cronbach’s alpha for all dimensions of the instrument separately. Three (7%) studies reported Cronbach’s alpha for the total scale: QOLS [81], MAPP-QOL [111], EUROHIS-QoL-8 [39]. And three (7%) studies reported Cronbach’s alpha for the component summary scores: SF-36 [58] and SF-12 [71, 72]. Twelve (28%) studies only reported Cronbach’s alpha for the total scale, even though the instruments do not provide a total scale score: SF-36 [57, 62–64], SF-12 [65, 66, 68–70, 73], and WHOQOL-BREF [25, 74]. None of these studies reporting Cronbach’s alpha only for total scales were validation studies. Three studies evaluated selected dimensions of the total scale, regarding the instruments RAND 36-item Health Survey (RAND-36) [87] and WHOQOL-BREF [25, 37].

**Table 4:** Psychometric evaluations of included instruments, reported according to COSMIN

Instrument	Measurement timepoint/period	Reliability [Reference to study evaluated in]			Validity [Reference to study evaluated in]			Responsiveness [Reference to study evaluated in]
		Internal consistency	Reliability	Measurement error	Content validity	Structural validity	Hypotheses testing	Responsiveness
Short form 36-item Health survey (SF-36)	Pregnancy	√ [57, 58, 61, 63, 110]				√ [41]		
	Postpartum	√ [40, 59, 60, 62, 64]		√ [40]				√ [40]
RAND 36-item Health Survey (RAND-36)	Pregnancy + postpartum	√ [87]^a^						
Short Form 12-item Health Survey (SF-12)	Pregnancy	√ [66, 69–71, 73]				√ [69]		√ [69]
	Postpartum	√ [65, 72]				√ [67, 72]	√ [72]	
	Pregnancy + postpartum	√ [68]	√ [68]					
Short Form 8-item Health Survey (SF-8)	Pregnancy	√ [91]						
World Health Organization Quality of Life Questionnaire Breif-version (WHOQOL-BREF)	Pregnancy	√ [45, 74, 75] [37]^b^				√ [37] ^b^		
	Postpartum	√ [38, 55, 76]				√ [38, 55]	√ [38]	
	Pregnancy + postpartum	√ [25^c^, 77]				√ [25]^c^	√ [25]^c^	√ [25]^c^
Quality of Life Scale (QOLS)	Postpartum	√ [81]						
Nottingham Health Profile (NHP)	Postpartum	√ [51]				√ [51]		√ [51]
EUROHIS-QoL-8		√ [39]						
EQ-5D-3L + EQ VAS	Postpartum		√ [80]	√ [40]		√ [80, 40]		√ [80, 40]
Patient Reported Outcomes Measurement Information System 43 (PROMIS-43)	Pregnancy	√ [104]						
Patient Reported Outcomes Measurement Information System Global Short Form (PROMIS GSF)	Pregnancy	√ [53]				√ [53]		
	Pregnancy + postpartum	√ [54]				√ [54]	√ [54]	
	Pregnancy + postpartum (9-item version)	√ [54]				√ [54]	√ [54]	√ [54]
Duke Health Profile (DUKE)	Pregnancy + postpartum					√ [109]		√ [109]
Maternal Quality of Life Index (M-QLI)	Pregnancy + postpartum	√ [110]						
Mother Generated Index (MGI)	Pregnancy (Antenatal version)					√ [43, 44]		
	Postpartum (Postnatal version)				√ [56]	√ [42, 44, 46, 78]	√ [42]	
Quality of life Gravidarum (QOL-GRAV)	Pregnancy	√ [45, 47, 79]	√ [47]		√ [47, 79]	√ [45, 47]		√ [45]
Maternal Postpartum Quality of Life Questionnaire (MAPP-QOL)	Postpartum	√ [52, 111]	√ [52]		√ [52]	√ [52, 111]	√ [111]	√ [111]
Rural postpartum quality of life (RPQOL)	Postpartum	√ [112]	√ [112]			√ [112]		
Postpartum Quality of Life (PQOL)	Postpartum	√ [49, 50]	√ [49, 50]		√ [49, 50]	√ [48–50]	√ [49]	
Short Form Postpartum Quality of Life Questionnaire (SF-PQOL)	Postpartum	√ [48]	√ [48]			√ [48]		

Cross-cultural validity, criterion validity and interpretability are not included in the table as no studies reported on these properties

^a^This study evaluated one of eight subscales: social functioning

^b^This study evaluated three of four domains: physical health, psychological, and social relationships

^c^This study evaluated two of four domains: physical health and social relationships

Structural validity was the second most commonly reported psychometric property, given in 27 studies (50.9%) and evaluated for all instruments except six: Short Form 8-item Health Survey (SF-8), RAND-36, QOLS, EUROHIS-QoL-8, Patient Reported Outcomes Measurement Information System 43 (PROMIS-43), and Maternal Quality of Life Index (M-QLI). Of these studies, eight (30%) measured correlation with other QoL instruments [42, 45, 46, 48, 49, 51–53], seven (26%) studies measured correlation between two or more dimensions in the instrument [37, 43, 44, 46, 52, 69, 109], five (19%) studies measured correlation between known-groups [25, 38, 40, 55, 111], and one (4%) study correlation between timepoints [78]. Ten (37%) studies reported exploratory factor analyses, confirmatory factor analyses and/or Rasch analyses [41, 47–50, 52, 54, 67, 72, 112]. Of the 27 studies reporting structural validity, 16 were validation studies [40–55], six were longitudinal [25, 69, 78, 80, 109, 111] and five were cross-sectional [37, 38, 67, 72, 112].

As shown in Table 4, content validity was measured in six (11.3%) studies. These studies addressed the period specific instruments MGI, QOL-GRAV, MAPP-QOL and POQL and all concerned women. Five of these studies were validation studies, four were conducted in the postpartum period, and two during pregnancy. Three studies were conducted in Iran, while the remaining three were from China, Brazil, and USA. None of the included studies provided data on content validity for any of the generic instruments.

Among the four studies that sampled couples, only three studies assessed psychometric properties for fathers and WHOQOL-BREF was the only instrument evaluated. In two studies [25, 37] Cronbach’s alpha was estimated for fathers separately from mothers, and in the third study [38] it was assessed for fathers and mothers together. Cronbach’s alpha was measured for each subscale of WHOQOL-BREF separately [37, 38], but in one study for all subscales together [25]. In addition, all three studies measured structural validity, correlation between dimensions of the instrument [37, 38] and between known-groups [25], on fathers alone. Cronbach’s alpha and structural validity were assessed in both pregnancy and the postpartum period.

We found that none of the 19 instruments had been evaluated for all nine psychometric properties recommended by COSMIN [15]. Further, none of the included studies reported on interpretability and cross-cultural validity. All in all, there was limited evidence on the psychometric properties of SF-8, RAND-36, QOLS, EUROHIS-QoL-8, PROMIS-43, and M-QLI. SF-36 was the most used instrument, but there was sparse evidence on Cronbach’s alpha for all scales of the instrument, as well as on other psychometric properties. A few instruments were evaluated in two or more studies and on three or more psychometric properties: EQ-5D-3L, PROMIS-GSF, MGI, and MAPP-QOL. Based on the number of reported psychometric properties in multiple studies, there was most information on SF-12, WHOQOL-BREF, QOL-GRAV, and PQOL. These instruments were all primarily assessed in Asian countries, but SF-12 was also assessed in some studies from Europe, North America, and Oceania. WHOQOL-BREF was also assessed in Europe, Africa, and Oceania, and QOL-GRAV also in Europe.

## Discussion

As one of the first reviews to summarize research on the psychometric properties of instruments used to measure QoL in parents during pregnancy or the postpartum period, our study provides valuable information for both practice and further research. We identified 53 studies which described 19 QoL instruments, of which none were evaluated on all nine psychometric properties recommended by COSMIN [15]. Interestingly, 83% of the studies were conducted in the last decade, with a pattern of increasing numbers of studies on evaluations of QoL instruments’ psychometric properties during the last few years. This suggests there is increasing interest in evaluations of QoL instruments for this target group and in a few years’ time there may be sufficient evidence on the instrument’s reliability and validity to undertake a thorough systematic review on the topic.

The most commonly measured psychometric properties were internal consistency and structural validity, which are part of the internal structure of an instrument. Internal structure refers to the relatedness of items in an instrument and is important to detect and determine items relevant for a scale or subscale. Evidence of structural validity, or unidimensionality, of a scale or subscale is a prerequisite to interpret measures of internal consistency [13]. Instruments with domains that make up a subscale score are assumed to represent a construct and is thereby considered a separate measure. Psychometric properties of these instruments should therefore be evaluated for each domain [13, 14], something several of the included studies failed to do in measures of Cronbach’s alpha. Multiple studies measured Cronbach’s alpha for the total scale, despite no evidence of a total score for the instrument. This typically concerned SF-36 and SF-12, which were two of the most used instruments. Thus, the constructs of these instruments were not properly evaluated. Encouragingly, the validation studies reported Cronbach’s alpha on all applicable domains with subscale scores.

Structural validity, the second most evaluated property, was measured for the majority of the identified instruments. However, only one third of the studies measuring structural validity were done by exploratory factor analyses, confirmatory factor analyses and/or Rasch analysis, as recommended by COSMIN [13, 17]. Evidently, these measures applied the specific instruments in larger amount than the generic instruments. The remaining analyses were mainly measured by variations of correlations that tests the construct of an instrument [5, 16]. However, few of these studies using correlations provided clear hypotheses for the correlations, making it difficult to ascertain whether the results are in accordance with the hypotheses or not [13], or if hypotheses were used at all. Consequently, there seems be several quality issues related to the measures of structural validity of the instruments identified in the present study. However, the extensive reporting on structural validity and internal consistency found in the present study is useful to understand the internal structure of the instruments. For some of the most evaluated instruments in our review—WHOQOL-BREF, QOL-GRAV, MAPP-QOL and PQOL—there is generous data on structural validity and internal consistency. Studies with this data could be assessed for methodological quality and systematically synthesized in a systematic review. For most of the other instruments, however, there is limited or insufficient evidence on their internal structure, and there is a need for further systematic evaluation in primary validation studies.

The remaining psychometric properties—reliability, measurement error, content validity, hypotheses testing, and responsiveness—were only reported in a few of the included studies. Cross-cultural validity, criterion validity and interpretability were not reported at all. Specifically, we lack evidence on the identified instruments’ responsiveness and reliability, which give information on the repeatability and ability to detect changes between timepoints and groups [5, 13]. Parental QoL and health status change throughout pregnancy and the first year postpartum [18–21, 23, 29, 113]. Therefore, evidence on the responsiveness and reliability of QoL instruments for this target group is needed. In addition, we lack evidence on content validity, only reported for some of the specific instruments. Content validity is considered the most important property because the items of the instrument must be relevant, comprehensive, and comprehensible to the construct of interest and the target group [13]. Most of the identified instruments in the present study included psychological, physical, and social dimensions, which are important aspects of the QoL concept [5]. Due to the ambiguity of QoL as a concept [1–3], and the specific changes and impact on health during pregnancy and the postpartum period for both mothers and fathers, there is a need to gain evidence on content validity for this specific target group. The insufficient reporting on content validity, especially for the generic instruments, is a significant weakness for the instrument’s validity to the population of parents in the pregnant and postpartum population.

The findings of our review show that there is limited evidence on psychometric properties of instruments used to measure QoL in fathers. Only three of the 53 studies reported psychometric properties of a QoL instrument used on fathers, and all used WHOQOL-BREF. Moreover, only a few of the recommended psychometric properties [15] of WHOQOL-BREF were evaluated. Consequently, evidence on appropriate and useful QoL instruments for fathers in the pregnant and postpartum period is nearly non-existent to researchers. During the pregnancy and postpartum period, fathers experience health challenges relevant for their QoL status [23, 29]. To gain useful and believable knowledge on fathers’ QoL in this crucial period of life, we need to use validated instruments that are appropriate to this target group [5]. Using instruments that are not validated for its intended purpose may lead to wrong conclusions, due to imprecise and biased results [17].

Nearly 50% of the included studies were conducted in Asia, with a high number of studies from Iran and China. In addition, the instruments that seemed to be most evaluated, were to a great extent assessed in Asia. Cultural conditions vary between countries, and the meaning or importance of a measurement may not be the same in different cultures [5, 14]. Also, aspects related to pregnancy and the postpartum period are perceived differently from culture to culture [114–116]. WHOs definition of QoL embraces the cross-cultural importance of the perception of QoL: “individuals’ perceptions … in the context of the culture and value-systems” [4]. Therefore, the cultural adaptation of a QoL instrument is of importance when evaluating its validity. Our review shows that there is a need for further knowledge on the instruments’ validity, reliability, and responsiveness, in all countries, but especially in countries outside of Asia.

### Implications for practice and research

The findings of our review are primarily useful to the research field, as further research on psychometric properties of the identified instruments is strongly needed. However, our findings may be useful for clinical practice, as it provides descriptive information of the characteristics and psychometric evaluations available for QoL instruments used in parents during pregnancy and the postpartum period. Increased use of the more thoroughly evaluated instruments, could lead to more precise results that are more relevant for this target group [17]. Unfortunately, we cannot provide recommendations on which instrument(s) to use in clinical practice, as this would have required quality assessments of the included studies and synthesis of results across studies. Additionally, we find that the evidence on the psychometric properties of instruments used to measure QoL in parents during pregnancy or the postpartum period is thus far too scarce to recommend conducting a full systematic review.

Further research on psychometric properties of QoL instruments for parents in pregnancy and the postpartum period should be designed as validation studies or primary studies. This is needed to provide sufficient evidence on the instrument’s appropriateness within this specific context and study population. There is a crucial need for evaluations of content validity of QoL instruments used in and developed for this target group. Increased evidence on psychometric evaluations from countries and continents outside of Asia are necessary to determine the cultural aspects of QoL instruments during this period of life. Additionally, our recommendations include that future research should focus on fathers, internal consistency measured by the instruments’ unidimensionality, more thorough evaluations of the instruments’ structural validity and internal consistency, and increased measures of reliability and responsiveness.

### Strengths and limitations

The strengths of our study include the systematic approach with a comprehensive literature search, dual screening and data extraction, and extensive examination of included studies. We included studies conducted on the general population of both mothers and fathers, during both pregnancy and the postpartum period. While this broad approach is in accordance with the aim of scoping reviews [30], it limits the possibility to go into details on e.g. populations and life periods. Due to the inclusion criterion about reporting of one or more psychometric properties, we did not include QoL instruments used in studies *without* measures of psychometric properties. It is possible that QoL instruments with no psychometric evaluation are being used in research. Although unlikely, there is also a possibility that relevant studies may have been excluded during the screening phases, due to missing information in the abstract, or that our search strategy did not detect relevant studies.

Our understanding of QoL was in line with the definition from WHO [4]. Thus, we did not include interrelated concepts with QoL, such as well-being, satisfaction with life and health status, in the search strategy. This strengthens our study by providing a clear, but broad, definition of QoL. The search strategy in our study included overall search terms on psychometric properties, such as validity and reliability. Inclusion of specific search terms of all psychometric properties, such as content validity and cross-cultural validity, could have identified additional studies. In a future systematic review on evaluation of psychometric properties in QoL instruments, it may be beneficial including such specific search terms.

In the present study we did not explore if the identified instruments originally were developed based on reflective or formative models, which is important for the understanding and evaluation of psychometric properties for each instrument [5, 13]. Consequently, our understanding of the instruments’ construct is based on how the scales of the identified instruments are reported to be scored. This perspective may be useful in methodological quality assessments of a future systematic review. Furthermore, we have chosen COSMIN’s categorization of psychometric properties [15], which may be inconsistent with other frameworks for psychometric properties. Recommendations and interpretations may change depending on choice of categorization of psychometric properties, and when conducting a more thorough evaluation of the psychometric measures and the methodological limitations of the included studies.

## Conclusions

The findings of our review show that the QoL instruments most commonly evaluated for one or more psychometric properties, on parents during pregnancy or the postpartum period, are SF-36, SF-12 and WHOQOL-BREF for generic QoL instruments, and MGI, QOL-GRAV and PQOL for specific QoL instruments. However, the four instruments with the most extensive information on psychometric properties are SF-12, WHOQOL-BREF, QOL-GRAV, and PQOL. We find that there is insufficient evidence on all psychometric properties, although there is extensive reporting on internal consistency and structural validity. Thus far, the evidence is too scarce to conduct a full systematic review on this topic. Rather, there is a need for validation studies and primary studies on the validity, reliability, responsiveness, and interpretability of QoL instruments in parents during pregnancy and postpartum period, in particular for fathers and partners.

## Supplementary Information

**Additional file 1**. Complete search strategy.

## Data Availability

Full data charting matrix is available from the corresponding author upon request.

## Abbreviations

QoL: Quality of Life
WHO: World Health Organization
COSMIN: Consensus-based standards for the selection of health measurement instruments
PRISMA-ScR: Preferred reporting items for systematic reviews and meta-analyses extension for scoping reviews
