# Supplementary material for: Quality of Life instruments and their psychometric properties for use in parents during pregnancy and the postpartum period: a systematic scoping review
Source: Health Qual Life Outcomes. 2022 Jul 9;20:107. doi: 10.1186/s12955-022-02011-y (PMC9271249; doi:10.1186/s12955-022-02011-y)
Supplement: Supplementary file 1 — Additional file 1. Complete search strategy. [file 12955_2022_2011_MOESM1_ESM.docx]

## **Additional file 1**

Additional file 1: Complete search strategy for all databases

| Database | Search terms |
| --- | --- |
| MEDLINE | (exp Parents OR parent* OR mother* OR exp Mothers OR exp Fathers OR father* OR maternal OR paternal OR woman OR women OR man OR men) AND (postpartum OR exp Postpartum Period OR post partum OR postnatal OR exp Postnatal Care OR post natal OR prenatal OR prepartum OR peripartum OR exp Peripartum Period OR perinatal OR exp Perinatal Care OR puerperium OR antenatal OR antepartum OR pregnan* OR exp Pregnancy) AND (quality of life OR exp Quality of Life OR HRQOL) AND (scale OR instrument OR psychometry OR psychometrics OR exp Psychometrics OR measurement* OR questionnaire* OR exp Surveys and Questionnaires OR assessment* OR reliability OR validity) |
| EMBASE | (parent* OR exp parent OR mother* OR exp mother OR father* OR exp father OR maternal OR paternal OR women OR woman OR man OR men) AND (postpartum OR post partum OR exp postnatal care OR postnatal OR post natal OR exp prenatal period OR exp prenatal care OR prenatal OR prepartum OR peripartum OR exp perinatal care OR exp perinatal period OR perinatal OR puerperium OR exp puerperium OR antenatal OR antepartum OR pregnan* OR exp pregnancy) AND (quality of life OR exp quality of life OR HRQOL) AND (scale* OR instrument* OR psychometry OR exp psychometry OR psychometrics OR measurement* OR exp questionnaire OR questionnaire* OR assessment* OR exp reliability OR reliability OR validity OR exp validity) |
| PsychINFO | (parent* OR exp Parents OR mother* OR exp Mothers OR father* OR exp Fathers OR maternal OR paternal OR woman OR women OR man OR men) AND (postpartum OR post partum OR exp Postnatal Period OR postnatal OR post natal OR exp Prenatal Care OR prenatal OR prepartum OR peripartum OR exp Perinatal Period OR perinatal OR puerperium OR antenatal OR exp Antepartum Period OR antepartum OR pregnan* OR exp Pregnancy OR pregnancy) AND (quality of life OR exp Quality of Life OR HRQOL) AND (scale* OR instrument* OR psychometry OR psychometrics OR exp Psychometrics OR measurement* OR exp Questionnaires OR questionnaire* OR assessment* OR exp Test Reliability OR reliability OR validity OR exp Test Validity) |
| CINAHL | (parent* OR MH Parents+ OR mother* OR MH Mothers+ OR father* OR MH Fathers+ OR maternal OR paternal OR women OR woman OR men OR man) AND (postpartum OR post partum OR MH Postnatal Period+ OR MH Postnatal Care+ OR postnatal OR post natal OR prenatal OR MH Prenatal Care OR prepartum OR peripartum OR MH Perinatal Care OR perinatal OR MH Puerperium OR puerperium OR antenatal OR antepartum OR pregnan* OR MH "Pregnancy+) AND (MH Quality of Life+ OR quality of life OR HRQOL) AND (scale* OR instrument* psychometry OR MH Psychometrics OR psychometrics OR measurement* OR questionnaire* OR assessment* OR MH Reliability+ OR reliability OR MH Validity+ OR validity) |
| HaPI | (parent* OR mother* OR father* OR maternal OR paternal OR woman OR women OR man OR men) AND (postpartum OR post partum OR postnatal OR post natal OR prenatal OR prepartum OR peripartum OR perinatal OR puerperium OR antenatal OR antepartum OR pregnan*) AND (quality of life OR HRQOL) AND (scale* OR instrument* OR psychometry OR psychometrics OR measurement* OR questionnaire* OR assessment* OR reliability OR validity) |
